# Determinants of Diabetic Peripheral Neuropathy and Their Clinical Significance: A Retrospective Cohort Study

**DOI:** 10.3389/fendo.2022.934020

**Published:** 2022-07-26

**Authors:** Yifan Cheng, Wen Cao, Junzhe Zhang, Jiabin Wang, Xiang Liu, Qianqian Wu, Qingxia Lin

**Affiliations:** ^1^ Department of Neurology, Center for Rehabilitation Medicine, Zhejiang Provincial People’s Hospital, Affiliated People’s Hospital, Hangzhou Medical College, Hangzhou, China; ^2^ Department of Neurology, The Third Hospital of Peking University, Beijing, China; ^3^ Department of Orthopaedic Surgery, The Third Hospital of Hebei Medical University, Shijiazhuang, China; ^4^ Department of Neurology, Hebei Medical University, Shijiazhuang, China; ^5^ Department of Geriatrics, Taizhou Hospital of Zhejiang Province Affiliated to Wenzhou Medical University, Taizhou, China; ^6^ Department of Psychiatry, First Affiliated Hospital of Wenzhou Medical University, Wenzhou, China

**Keywords:** type-2 diabetes mellitus, diabetic peripheral neuropathy, determinants, clinical significance, prevalence, risk factor

## Abstract

**Background:**

In this study, we investigated the epidemiological characteristics and predictors of diabetic peripheral neuropathy (DPN) in adult patients with type 2 diabetes mellitus (DM).

**Methods:**

The study was designed as a retrospective cohort trial at the First Affiliated Hospital of Wenzhou Medical University. From January 2017 to December 2020, a total of 1,262 patients with DM were enrolled to assess the risk factors for DPN. The patients were divided into two groups (DPN group and non-DPN group). The Mann–Whitney *U* test or *t*-test, receiver operating characteristic (ROC) analyses, univariate chi-square analyses, and multiple logistic regression analyses were used to analyze the adjusted predictors of DPN.

**Results:**

The overall prevalence of DPN in DM patients was 72.7% (*n* = 793/1,091). Multivariate analysis revealed that age > 66 years (odds ratio [OR], 2.647; 95% confidence interval [CI] 1.469–4.770; *p* = 0.002), history of hypertension (OR, 1.829; 95% CI 1.146–2.920; *p* = 0.011), neutrophil (NE) levels exceeding 4.0 × 10^9^/L (OR 0.256; 95% CI 0.162–0.405; *p* = 0.001), lymphocyte (LY) levels over 3.0 × 10^9^/L (OR 7.173; 95% CI 4.258–12.086; *p* = 0.000), HbA1c > 7.7% (OR 3.151; 95% CI 1.959–5.068; *p* = 0.000), and FT3 > 4.4 pmol/L (OR 0.417; 95% CI 0.263–0.662; *p* = 0.000) were six significant predictive factors for the prevalence of DPN.

**Conclusions:**

High levels of LY, HbA1c, history of hypertension, and > 66 years of age increase the risk of DPN in adult patients with DM, while high levels of NE and FT3 were protective factors of DPN. Thus, the prediction of DPN can significantly be improved by identifying older patients over the age of 66 and history of hypertension, as well as establishing the biochemical cutoff values of NE, LY, HbA1c, and FT3.

## Introduction

Diabetes mellitus (DM) is a chronic metabolic disease associated with hyperglycemia owing to impaired insulin secretion, insulin activity, or both (1). High levels of blood glucose can cause damage to all organs of the body, including the cardiovascular system, eyes, kidneys, as well as the nervous system (2). The pathogenesis of diabetes is very complex, and a large number of studies are under way. Some studies have found that anemia, which could lead to tissue hypoxia, interferes with the diagnosis and management of diabetes because it affects pathological examination and drugs (3). In 2019, 488 million adults aged 20–99 years old (9.5%) live with diabetes, worldwide. The high cost of the treatment of diabetes and the loss of the ability to work normally as a result of diabetes are a burden on society (4).

Diabetic peripheral neuropathy (DPN) is the most widely recognized complication of DM (5). At present, DPN is divided into two clinical types: typical DPN (DSPN) and atypical DPNs. Symptoms or signs of DSPN may include the following: decreased sensation; positive neurosensory symptoms (such as “sleep numbness”, tingling, burning, or pain), mainly in the toes, feet, or legs; reduced distal sensory symmetry; and absent ankle reflexes. In the exploration of DPN, there are many previous studies suggesting that diabetes is closely related to other complications. Cardiovascular autonomic neuropathy is positively correlated with declined urinary albumin excretion rate and cardiac function in patients with diabetes (6). In patients with atypical DNPs, pain and autonomic and neuromorphologic abnormalities may be present (7). It mainly manifests as peripheral body pain (8), cardiovascular system damage (9), sudomotor dysfunction (10), bladder dysfunction, and erectile dysfunction (11). In a prospective study with long-term follow-up, the prevalence of DPN increased from 7.5% at the beginning of DM to 45% after 25 years with DM (12). The high prevalence of DPN is a major cause of death and disability resulting from diabetes, including recurrent limb infections, ulcers, and even progression to amputation. One study reported an 11% cumulative risk of lower limb amputation 25 years after the diagnosis of diabetes (13).

The pathogenesis of DPN has not been clearly elucidated. Some scholars have suggested that DPN can be attributed to chronic exposure to hyperglycemia and cardiovascular risk covariates (7). The existing examination and diagnosis methods, such as nerve biopsy, skin biopsy, and flare reaction, are difficult to operate and are not acceptable to patients. Due to the lack of treatments that target the underlying nerve damage, screening for laboratory indicators for DPN is critical in clinical practice, as it may detect the earliest stages of neuropathy, enabling early intervention (14). The objective of this retrospective study was to identify independent risk factors for DPN in DM patients and to provide insights for clinical diagnosis and management.

## Patients and Methods

### Patients

**Figure 1:**
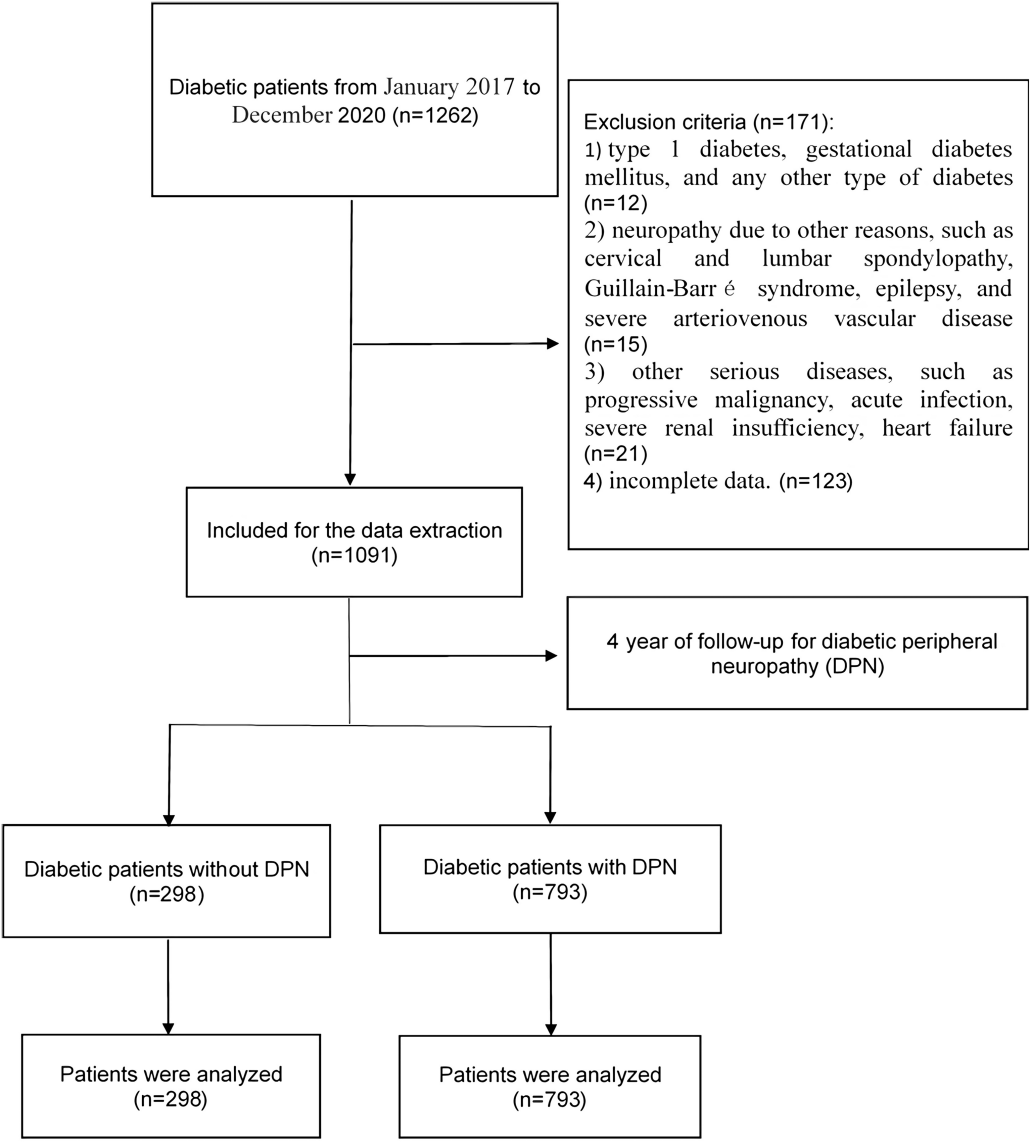
The flow chart for the selection of study participants.

Information on diabetic patients who underwent treatment for diabetes at our hospital from January 2017 to December 2020 was extracted from a retrospective database. A total of 1,262 cases were reviewed, and 1,091 were analyzed (**Figure 1**). The exclusion criteria were as follows: (1) type 1 diabetes, gestational DM, and any other type of diabetes; (2) neuropathy due to other reasons, such as cervical and lumbar spondylopathy, Guillain–Barré syndrome, epilepsy, and severe arteriovenous vascular disease; (3) other serious diseases, such as progressive malignancy, acute infection, severe renal insufficiency, and heart failure; (4) several medications (thyroid hormones preparations, methimazole, propylthiouracil, amiodarone, lithium carbonate, corticosteroids, biotin, etc.) that are able to affect thyroid function or to influence thyroid hormone levels; (5) incomplete data; and (6) several medications (thyroid hormones preparations, methimazole, propylthiouracil, amiodarone, lithium carbonate, corticosteroids, biotin, etc.) that are able to affect thyroid function or to influence thyroid hormone levels were excluded.. The patients in our study were divided into 2 groups: DM patients who developed DPN and DM patients without DPN. The study was approved by the institutional ethics committee of the First Affiliated Hospital of Wenzhou Medical University, and all procedures were performed in accordance with the principles outlined in the Declaration of Helsinki. Informed consent was obtained from all patients.

### Definition and Detection of DPN

DPN is defined as clinical and(or) electrophysiological evidence of the definite presence of peripheral neuropathy in patients with diabetes. Other causes of peripheral neuropathy should also be ruled out. All T2DM patients underwent a complete neurological examination by experienced neurologists. Symptoms were assessed using the neuropathy disability score based on the examination of ankle reflexes, vibration, pinprick sensation, and temperature sensation. Nerve conduction studys (NCS) were performed on each patient by trained physicians. During the test, patients remained calm and relaxed, and local skin temperature was kept constant.

The diagnosis met the following criteria: (1) a clear history of diabetes or at least evidence of abnormal glucose metabolism; (2) neuropathy that occurs at or after the diagnosis of diabetes; (3) the clinical symptoms and signs were consistent with those of DPN; (4) if there are 2 or more abnormalities in the following 5 tests: (a) temperature sensing anomaly, (b) Semmes–Weinstein monofilament trial: decline or absence of sensation in the foot, (c) abnormal vibration sensation, (d) absence of ankle reflex, and (e) nerve conduction velocity decreased by 2 or more factors; (5) other neuropathy, such as cervical and lumbar lesions, was excluded.

### Data Acquisition and Variables of Interest

The data came from six aspects: demographics, blood indicators, lipid metabolism, glucose metabolism, renal function, and thyroid hormones. The demographic data included age, gender, residence, body mass index (BMI), cigarette smoking, and alcohol consumption. The blood indicators included WBC, RBC, and platelet counts; neutrophil count; mononuclear cell count; and lymphocyte count. The lipid metabolism data included total cholesterol (TC), triglyceride (TG), high-density lipoprotein (HDL), and low-density lipoprotein (LDL). The glucose metabolism included HbA1c and fasting blood glucose (FBG). The renal function included creatinine, urea, and serum uric acid. The thyroid hormones included thyroid-stimulating hormone (TSH), free triiodothyronine 3 (FT3), and free thyroxine 4 (FT4). Associated laboratory variables were obtained within 24 h of admission. FT3, FT4, and TSH were tested by Beckman DXI800 (Beckman Coulter, Indiana, USA).

### Statistical Analysis

Statistical analyses were performed using SPSS version 25.0 (IBM Corp., Armonk, NY, USA). Continuous variables were presented as median, mean ± standard deviation (SD). Student’s *t*-test and Mann–Whitney *U* test were performed to compare continuous variables between DPN and non-DPN groups according to the homogeneity of variance test and normality test. For the continuous variables with statistical significance, receiver operating characteristic (ROC) analyses were performed to detect the optimum cutoff value, which was calculated by maximizing the sum of sensitivity and specificity in the ROC curve. Based on the cutoff value, continuous variables were converted into categorical variables before being subjected to logistic regression. Pearson chi-square test was used to determine the correlation between each classification variable and DPN. Predictors found to be significant in the single-factor analysis were subjected to stepwise multiple logistic regression analyses (backward LR) to screen for the adjusted factors. The odds ratio (OR) and 95% confidence interval (CI) were determined to evaluate the correlation magnitude between factors and DPN. *p* < 0.05 was considered to be statistically significant.

## Results

It is a retrospective single-center study involving a total of 1,262 patients with DM. **Figure 1** shows the flowchart that represents the procedures applying for screening the study participants. A total of 1,262 diabetic patients were admitted to our institute. Among them, 12 patients suffered from type 1 diabetes, gestational DM, and any other type of diabetes; 15 patients had neuropathy due to other reasons, such as cervical and lumbar spondylopathy, Guillain–Barré syndrome, epilepsy, and severe arteriovenous vascular disease; 21 patients had other serious diseases, such as progressive malignancy, acute infection, severe renal insufficiency, and heart failure; 123 patients had incomplete data before admission. A total of 171 patients were excluded due to incomplete data. A total of 1,091 patients were eventually analyzed in this study; 678 (62%) were men and 413 (38%) were women. Their average age and BMI were 54.79 ± 12.32 years old vs. 60.40 ± 11.53 years old and 24.56 ± 3.76 kg/m^2^ vs. 24.10 ± 3.30 kg/m^2^, respectively. The prevalence of diabetic patients with DPN is 72.7%.

As shown in **Table 1**, thirteen continuous variables were compared between the DPN group and the non-DPN group. Five continuous variables including age, NE, LY, HbA1c, and FT3 were significantly different between the two groups. The optimum cutoff values for quantitative data related to DNP were determined by ROC curve analysis. **Table 2** shows the area under the curve and the optimum cutoff values for the five continuous variables. The optimum cutoff values for age, NE, LY, HbA1c, and FT3 were 66.0 years, 4.0 × 10^9^/L, 3.0 × 10^9^/L, 7.7%, and 4.4 pmol/L, respectively.

**Table 1:** Comparison of general information and biochemical indicators between non-DPN group and DPN group.

Variables	Non-DPN group (mean ± SD) (n=298)	DPN group (mean ± SD) (n=793)	P-value
Age (years)	54.79 ± 12.32	60.40 ± 11.53	0.000*
BMI (kg/m2)	24.56 ± 3.76	24.10 ± 3.30	0.137
Gender (males)	167 (56.0)	511 (64.4)	0.011*
Age (>66 years)	39 (13.1)	245 (32.3)	0.000*
Smoking	78 (26.2)	266 (33.5)	0.020*
Hypertension	113 (37.9)	465 (58.6)	0.000*
Hyperlipidemia	87 (30.4)	236 (29.8)	0.835
TC (mmol/L)	4.86 ± 1.31	5.83 ± 22.36	0.084
TG (mmol/L)	1.98 ± 1.92	1.91 ± 1.60	0.091
HDL (mmol/L)	1.02 ± 0.40	1.04 ± 0.31	0.196
LDL (mmol/L)	2.65 ± 0.91	3.12 ± 11.80	0.067
SUA (μmol/L)	314.77 ± 91.04	329.60 ± 100.98	0.103
NE (*109/L)	4.11 ± 1.61	3.94 ± 2.11	0.003*
LY (*109/L)	2.14 ± 0.66	2.94 ± 1.15	0.000*
HbA1c(%)	9.08 ± 2.83	9.39 ± 2.60	0.030*
TSH (mU/L)	1.69 ± 1.32	1.75 ± 2.43	0.805
FT3(pmol/L)	4.80 ± 0.65	4.60 ± 1.44	0.000*
FT4 (pmol/L)	11.19 ± 1.97	11.28 ± 2.10	0.251
FT3/FT4	0.441 ± 0.011	0.443 ± 0.214	0.969

**Table 2:** The ROC curve analysis of continuous variables with statistical significance.

Variable	Cut-off value	Area under the curve (95% CI)	Sensitivity	Specificity	P-value
Age (years)	66.0	0.630 (0.593-0.668)	32.8%	88.6%	0.000
NE (*10^9^/L)	4.0	0.560 (0.520-0.601)	52.1%	65.4%	0.003
LY (*10^9^/L)	3.0	0.720 (0.687-0.753)	42.3%	91.5%	0.000
HbA1c (%)	7.7	0.545 (0.503-0.586)	70.6%	43.2%	0.030
FT3 (pmol/L)	4.4	0.600 (0.563-0.637)	74.7%	44.4%	0.000

ROC receiver operating characteristic; CI confidence interval; NE neutrophil; LY lymphocyte; HbA1c Hemoglobin A1c; TSH thyroid stlmulating hormone; FT3 free triiodothyronine 3.

The demographic and laboratory indicators were evaluated by univariate analysis in **Table 3**. A total of eight factors (gender, age, smoking, hypertension, NE, LY, HbA1c, and FT3) were correlated with DPN. Fifty-six percent of patients were male in the DPN group, and 64.4% of patients were male in the non-DPN group. The proportion of patients >66 years old was higher in DM patients with DPN than in those without DPN (32.3% vs. 13.1%) (*p* < 0.001). More patients with DPN had histories of hypertension (*p* < 0.001) and smoking (*p* < 0.020), but not hyperlipidemia (*p* = 0.835). Other characteristics, including NE > 4.0 × 10^9^/L (*p* < 0.001), LY > 3.0 × 10^9^/L (*p* < 0.001), HbA1c > 7.7% (*p* < 0.001), and FT3 > 4.4 pmol/L (*p* < 0.001), were statistically significantly different between the two groups. Therefore, these eight factors were subjected to the multiple logistic regression analysis.

**Table 3:** Comparison of categorical variables between non-DPN group and DPN group.

Variables	Number (%) of patients in non-DPN group (n = 298)	Number (%) of patients in DPN group (n = 793)	P-value
NE (>4.0*10^9^/L)	155 (52.0)	64 (26.2)	0.000
LY (>3.0*10^9^/L)	28 (9.4)	311 (41.0)	0.000
HbA1c (>7.7%)	154 (56.8)	484 (70.0)	0.000
FT3 (>4.4pmol/L)	215 (73.6)	396 (54.5)	0.000

*Statistical significance.

DPN diabetic peripheral neuropathy; NE neutrophil; LY lymphocyte; HbA1c Hemoglobin A1c; FT3 free triiodothyronine 3.

The final variables of the multiple logistic regression analysis are shown in **Table 4**. There were six independent risk factors for DPN: >66 years of age (OR 2.647; 95% CI 1.469–4.770; *p* = 0.001), hypertension (OR 1.829; 95% CI 1.146–2.920; *p* = 0.011), NE > 4.0 × 10^9^/L (OR 0.256; 95% CI 0.162–0.405; *p* < 0.0001), LY > 3.0 × 10^9^/L (OR 7.173; 95% CI 4.258–12.086; *p* < 0.0001), HbA1c > 7.7% (OR 3.151; 95% CI 1.959–5.068; *p* < 0.0001), and FT3 > 4.4 pmol/L (OR 0.417; 95% CI 0.263–0.662; *p* < 0.0001). The Hosmer–Lemeshow test showed adequate fitness (*χ* ^2^ = 3.612; *p* = 0.890).

**Table 4:** Multivariate analysis (Multiple logistic regression analysis) of factors associated with diabetic peripheral neuropathy.

Variables	Odds ratio	95% CI	P-value
Age (>66 years)	2.647	1.469-4.770	0.001
Hypertension	1.829	1.146-2.920	0.011
NE (>4.0*10^9^/L)	0.256	0.162-0.405	0.000
LY (>3.0*10^9^/L)	7.173	4.258-12.086	0.000
HbA1c (>7.7%)	3.151	1.959-5.068	0.000
FT3 (>4.4pmol/L)	0.417	0.263-0.662	0.000

CI confidence interval; NE neutrophil; LY lymphocyte; HbA1c Hemoglobin A1c; FT3 free triiodothyronine 3.

## Discussion

In this study, a large-sample retrospective cohort was applied to address the epidemiologic characteristics of diabetic patients with DPN. We found that the overall prevalence of diabetic patients with DPN was 72.7%. Six predictive factors, namely, age (>66 years), hypertension, NE (>4.0 × 10^9^/L), LY (>3.0 × 10^9^/L), HbA1c (>7.7%), and FT3 (>4.4pmol/L), were independently correlated with DPN.

The overall prevalence of DPN among diabetic patients analyzed was 793/1,091 (72.7%). Our study result was similar to the studies reported from Iran (75.1%) and Italy (82%) (15, 16). However, our prevalence was higher than other studies reported from the USA, Malaysia, and Ethiopia where the prevalence were found to be 51%, 50.7%, and 53.6%, respectively (17, 18) and even higher than a study in Jordan, which was 39.5% (19). Besides the disparities in genetic predisposition, in healthcare qualities, and in study settings and designs, the high prevalence of DPN in diabetic patients indicates poor patient’s self-conception of symptom of DPN and deficits of patient’s education, which should be stressed in future medical service.

Age, as a predictor for DPN, has been reported previously (18, 20, 21). Patients > 66 years old were 2.65 times more likely to develop DPN than those < 66 years old. Three cross-sectional studies revealed that age > 40 years old and >50 years old were independent predictors for DPN (18, 21, 22). In the study of Abdissa et al. (18), patients > 40 years old and >50 years old were 4.57 times and 6.5 times more likely to develop DPN compared to patients whose age was less than 30 years old, respectively. DPN is a chronic complication needing time to develop, which may be the reason for this association between age and DPN.

HbA1c value was used to assess the quality of the glycemic control. According to the present study, there was a significant association between DPN and HbA1c > 7.7% (OR 3.151; 95% CI 1.959–5.068; *p* < 0.001). This association between HbA1c and DPN has been reported previously by Darivemula et al. (23), showing that compared to patients with normal HbA1c levels (<7%), those with high HbA1c (>7%) were 4.81 times more likely to develop DPN. One percent increase in HbA1c levels was positively related to microvascular rather than macrovascular complications (24). It is reported that high level of blood glucose is correlated with the severity of DPN (25).

Hypertension has been identified as a strong independent risk factor for DPN in patients with DM (26–32). Our finding is consistent with previous studies. In contrast, treatment of hypertension in patients with T2DM shows a significant reduction in the prevalence of DPN, which shows that vascular dysfunction plays an essential role in the pathogenesis of DPN. In the study of Ponirakis et al. (32), no effect on neuropathy hypertension was found in cases without diabetes. With respect to the underlying pathophysiology behind hypertension, loss of myogenic tone and vascular hypertrophy have been previously revealed in resistance vessels of diabetic patients with DPN (33), which was partially alleviated after improved glycemic control (34, 35). Further studies are needed to clarify the physiologic link between hypertension and DPN in diabetic patients.

LY showed the most powerful relationship with DPN in diabetic patients. Diabetic patients with LY >3.0 × 10^9^/L are 7.17 times (95% CI 4.258–12.086; *p* < 0.001) more prone to DPN than those with LY < 3.0 × 10^9^/L. Both T1DM and T2DM, as well as their complications, had been identified as inflammatory disease and a dysfunction status of the immune system (36, 37). White blood cell (WBC) count and its subtypes are inflammatory indicators. Neutrophils are closely associated with ongoing inflammation, and lymphocytes present the state of the immune system (38). A retrospective study conducted by Liu et al. (39) demonstrated that T2DM patients with higher neutrophil-to-lymphocyte ratio (NLR) levels are more likely to develop DPN. Accompanied by CD8+ T cells, NK cells play an essential role in the immune response to peripheral neuropathy (40), including not only their cytotoxicity, but also the selective pruning of damaged sprouting peripheral axons.

Moreover, our study has revealed two protective factors for DPN in diabetic patients. To the best of our knowledge, the present study is the first to identify NE (>4.0 × 10^9^/L) as a protective factor for DPN. Neutrophils are multifaceted cells acting either protectively or harmfully, depending on the specific disease condition, the activation state of neutrophils, and the body compartment affected (41). Besides their effects on tissue injury, neutrophils showed effects on tissue repair by releasing growth factors (42). A set of studies has found the relationship between neutrophils and DM. Elevated phagocytic activity of neutrophils had been revealed to be improved due to treatment of diabetic foot infections (43). Insulin can increase neutrophil count and function in diabetic patients following cardiac surgery (44, 45). Whether neutrophils alleviate or aggravate DPN calls for more studies.

Neutrophils are traditionally regarded as a simple infantry of the innate immune system, with a limited set of pro-inflammatory functions. It is clear that neutrophils are actually complex cells with a large number of special functions (46). In our study, we found that the increase of neutrophils may be a protective factor of DPN, which is not consistent with our usual cognition. Neutrophils play a variety of roles in acute viral infection. They limit virus replication and spread through phagocytosis, threshing, respiratory burst, secretion of cytokines, and release of extracellular traps of neutrophils, and activate adaptive immune response, thus playing a protective role in tissue damage (47). Studies have found that neutrophils have been observed to prevent and promote the establishment of infection, and neutrophils may affect the persistence of chronic parasites in a mouse model of leishmaniasis (48). The pathogenesis of DPN is still unknown. There is a question about whether this interesting finding in our study, contrary to previous perceptions, will further advance our understanding of DPN.

An FT3 level of more than 4.4 pmol/L showed a protective effect on DPN patients. In the study of Fernandez-Real et al., an intrinsic link between thyroid function tests and insulin resistance had been demonstrated (49). Sendi et al. conducted a multicenter cross-sectional study and found a significant association between DNP and comorbidities including thyroid diseases (50). However, Yuan et al. considered that FT3 or FT4 alone may not be the best predictor to truly reflect the integral alteration of free thyroid hormone metabolism, and they found that a low FT3/FT4 ratio level is an independent risk factor in euthyroid patients with three-vessel disease (51). Park et al. found that the high FT3/FT4 ratio is associated with insulin resistance, and the elevated FT3/FT4 ratio could be the result of adapting to adverse insulin resistance. Further studies are needed to investigate this relationship between DPN and FT3. In our study, male gender and history of smoking were not significantly associated with DPN in diabetic patients. In our study, we found that an FT3 level higher than 4.4 pmol/L has a protective effect on DPN. A large number of previous studies have also shown that FT3 has an important link to non-thyroid diseases. Hypothyroidism, including a decrease in FT3, has been found to be associated with nephrotic syndrome. This is considered to be a common feature of primary and secondary glomerular disease, with loss of protein in urine and increased urinary excretion of thyroid hormone and thyroxine-binding globulin (52). The level of plasma free thyroid hormone and FT3 decreased rapidly in acute critically ill patients, which was found to be related to the decrease of myocardial function. After administration of T3/T4, myocardial dysfunction was rapidly reversed (53). A meta-study of suicidal tendencies found that 2,807 participants were involved. The study found that patients with suicidal behavior had lower levels of FT3 than patients without suicidal behavior, and the average levels of FT3 and TT4 in patients with suicidal behavior were significantly lower than those without suicidal behavior (54). These studies focus more on the relationship between the reduction of FT3 and disease. Our results suggest that the increase of FT3 may be a protective factor, which may be an important research direction for us to further explore DPN in the future.

In the study of thyroid hormone and aging, more and more researchers pay attention to the important role of FT3/FT4. A study of 672 well-defined Italian subjects found that FT3 levels and the FT3/FT4 ratio decreased, while FT4 and TSH increased in an age-dependent manner (55). Another study recruited 593 well-defined Italian subjects, including 180 centenarians, the offspring of 276 centenarians, and 137 age-matched controls. Their results found a link between thyroid hormone levels and weakness in centenarians, which is the basis of the important role of thyroid in aging and longevity (56). Further studies found a possible correlation between the decrease of FT3/FT4 (an indirect marker of thyroxine deiodination disorder) and the debilitating state and survival rate of hospitalized elderly patients (57).

We acknowledge some limitations to our study. It involved only one institution, and a retrospective cohort study cannot imply cause and effect. Multicenter and prospective studies should be conducted to investigate the prevalence and risk factors of DPN in diabetic patients to confirm our findings. Nonetheless, a total of 1,091 patients, a relatively large simple capacity, were analyzed in our study. We performed 4 years of follow-up for DPN. Additionally, 13 continuous variables were evaluated by ROC curve analysis to identify the most sensitive cutoff value.

## Conclusion

We found that the prevalence of DPN in diabetic patients was 72.7%. Age (>66 years old), hypertension, NE (>4.0 × 10^9^/L), LY (>3.0 × 10^9^/L), HbA1c (>7.7%), and FT3 (>4.4 pmol/L) were six significant predictors for the occurrence of DPN. We hope that these factors are useful for the individualized assessment, risk stratification, and development of targeted prevention programs.

## Limitation

As a cross-sectional study, we can only preliminarily study the significance of some blood parameters in the first diagnosis of DPN patients. In the long course of disease of patients with DM, many influencing factors are not fully included. Current research lacks the most important variables related to the occurrence of DPN. This is also the limitation of our research.

## Data Availability Statement

The data analyzed during the current study is available from the corresponding author upon reasonable request.

## Ethics Statement

The studies involving human participants were reviewed and approved by the Ethical Decision Committee of the Research Administration at First Affiliated Hospital of Wenzhou Medical University. The patients/participants provided their written informed consent to participate in this study.

## Author Contributions

YC, WC, and JZ were responsible for data statistics and writing the paper. JW, XL, and QW collected data. QL provided resources and designed the study. All authors contributed to the article and approved the submitted version.

## Conflict of Interest

The authors declare that the research was conducted in the absence of any commercial or financial relationships that could be construed as a potential conflict of interest.

## Publisher’s Note

All claims expressed in this article are solely those of the authors and do not necessarily represent those of their affiliated organizations, or those of the publisher, the editors and the reviewers. Any product that may be evaluated in this article, or claim that may be made by its manufacturer, is not guaranteed or endorsed by the publisher.
