# Supplementary material for: Determinants of Diabetic Peripheral Neuropathy and Their Clinical Significance: A Retrospective Cohort Study
Source: Front Endocrinol (Lausanne). 2022 Jul 26;13:934020. doi: 10.3389/fendo.2022.934020 (PMC9360478; doi:10.3389/fendo.2022.934020)
Supplement: Supplementary file 1 [file Table_1.docx]

| **Supplementary material table 1** Multivariate analysis (Multiple logistic regression analysis) of factors associated with diabetic peripheral neuropathy | | | |
| --- | --- | --- | --- |
| **Variables** | **Odds ratio** | **95% CI** | **P-value** |
| Age | 1.053 | 1.036-1.069 | 0.00 |
| Sex | 1.595 | 1.079-2.358 | 0.02 |
| Smoke | 1.240 | 0.820-1.874 | 0.31 |
| Hypertension | 1.903 | 1.351-2.679 | 0.00 |
| NE | 0.841 | 0.749-0.944 | 0.00 |
| LY | 2.904 | 2.329-3.621 | 0.00 |
| HbA1c | 1.115 | 1.038-1.199 | 0.00 |
| FT3 | 0.891 | 0.793-1.000 | 0.05 |
| *CI* confidence interval; *NE* neutrophil; *LY* lymphocyte; *HbA1c* Hemoglobin A1c; *FT3* free thyroxine 3. | | | |
